# Implementation of Integrated Smart Construction Monitoring System Based on Point Cloud Data and IoT Technique

**DOI:** 10.3390/s25133997

**Published:** 2025-06-26

**Authors:** Ju-Yong Kim, Suhyun Kang, Jungmin Cho, Seungjin Jeong, Sanghee Kim, Youngje Sung, Byoungkil Lee, Gwang-Hee Kim

**Affiliations:** 1 Technical Research Center & Research Institute, Sunmoon Co., Ltd., Seoul 04130, Republic of Korea; wndyd9421@gmail.com; 2 School of Architecture, Yeungnam University, Gyeongsan-si 38541, Republic of Korea; yp043422@ynu.ac.kr; 3 Department of Civil Engineering, Kyonggi University, Suwon 16227, Republic of Korea; whqkrtk2@kyonggi.ac.kr (J.C.); jsj7629@kyonggi.ac.kr (S.J.); basil@kyonggi.ac.kr (B.L.); 4 Department of Architectural Engineering, Kyonggi University, Suwon 16227, Republic of Korea; sanghee0714@kyonggi.ac.kr; 5 Department of Electronic Engineering, Kyonggi University, Suwon 16227, Republic of Korea; yjsung@kyonggi.ac.kr

**Keywords:** point cloud data, worker positioning, concrete strength monitoring, construction safety, smart construction technology

## Abstract

This study presents an integrated smart construction monitoring system that combines point cloud data (PCD) from a 3D laser scanner with real-time IoT sensors and ultra-wideband (UWB) indoor positioning technology to enhance construction site safety and quality management. The system addresses the limitations of traditional BIM-based methods by leveraging high-precision PCD that accurately reflects actual site conditions. Field validation was conducted over 17 days at a residential construction site, focusing on two floors during concrete pouring. The concrete strength prediction model, based on the ASTM C1074 maturity method, achieved prediction accuracy within 1–2 MPa of measured values (e.g., predicted: 26.2 MPa vs. actual: 25.3 MPa at 14 days). The UWB-based worker localization system demonstrated a maximum positioning error of 1.44 m with 1 s update intervals, enabling real-time tracking of worker movements. Static accuracy tests showed localization errors of 0.80–0.94 m under clear line-of-sight and 1.14–1.26 m under partial non-line-of-sight. The integrated platform successfully combined PCD visualization with real-time sensor data, allowing construction managers to monitor concrete curing progress and worker safety simultaneously.

## 1. Introduction

The construction industry records the highest number of fatal accidents among all industries and maintains the highest fatality rates globally [1,2,3]. The disproportionately high rate of fatal accidents relative to the proportion of workers in the industry has led to industrial accident management in construction being consistently emphasized [4]. However, managing industrial accidents to prevent construction site incidents is far from an easy task. This is because construction sites are highly dynamic environments characterized by continuous changes in workers, machinery, and equipment, making prediction challenging. In particular, recent advancements in technology and the introduction of various equipment and innovative technologies have further increased this complexity. Consequently, managing industrial accidents has become even more challenging, with diverse risks present at construction sites. Safety managers must now consider a greater number of factors to perform thorough and efficient safety inspections.

Most construction accidents occur due to a multitude of interrelated factors [5]. Therefore, comprehensive elements must be considered for accident prevention, including worker safety management (e.g., PPE usage, access control to hazardous areas, worker health conditions, safety training), material quality management (e.g., concrete strength), structural safety management (e.g., deformation of formwork and steel structures), and work environment management (e.g., equipment usage monitoring, site cleanliness maintenance). Failure to monitor and address these factors in a timely manner can lead to fatal accidents. For instance, in January 2022, a tragic incident occurred at a high-rise apartment construction site in Korea, where the upper section of a 39-story building collapsed, resulting in worker fatalities and disappearances. According to the Korean Ministry of Land, Infrastructure, and Transport’s construction accident investigation report, the causes were identified as insufficient concrete strength and premature removal of temporary facilities (e.g., shoring), leading to reduced structural stability. Construction site accidents such as worker falls, structural collapses, and equipment collisions are diverse, with highly complex causes. Given that most accidents result from a combination of interconnected factors, it is difficult to ascertain independent causes for each incident, as demonstrated through quantitative research [6]. Therefore, preventing construction site accidents requires systematic management and continuous monitoring across various dimensions, including structural stability, construction quality, and worker management. Particularly during construction, comprehensive monitoring of both structural quality and worker safety is essential. Such monitoring plays a critical role in preventing accidents and fostering a safe working environment.

In recent years, advancements in sensor and Internet of Things (IoT) technologies have led to the proposal of automated methods for monitoring construction sites. Sensors are devices that detect changes in the physical environment and collect data. Devices applied for construction site monitoring include location sensors (e.g., Radio-Frequency Identification (RFID), Ultra-Wideband (UWB), Global Positioning System (GPS)), temperature sensors, Inertial Measurement Units (IMUs) (accelerometers, gyroscopes), and mapping sensors (e.g., 3D laser scanners, RGB cameras). Location sensors are primarily used to identify the real-time locations of workers and equipment, preventing access to hazardous zones and collisions [7]. According to quantitative research, location sensor technology has been shown to reduce worker safety incidents by an average of 45% [8]. Temperature sensors measure environmental temperatures to prevent heatstroke or monitor concrete curing temperatures [9]. Experimental results show that they can improve concrete strength prediction accuracy up to 87.5%. IoT and communication technologies, such as Cellular Communications (LTE, 5G), Bluetooth Low-Energy (BLE), Wi-Fi, and UWB, enhance safety by enabling real-time data collection and alert notifications [10]. Quantitative research has confirmed that the integrated application of these technologies results in a 63.8% reduction in site safety incidents. These studies confirm that individual sensing technologies are suitable tools for construction site monitoring. However, most existing research focuses on specific technologies and limited scopes, with insufficient studies addressing comprehensive monitoring for areas such as worker safety management and quality control.

Meanwhile, various studies have been conducted to integrate these sensing technologies with Building Information Modeling (BIM) for visualizing construction site conditions [11,12]. While BIM is an advantageous tool for design and construction management, data integration, and simulation, it has certain limitations when applied to real-time construction site monitoring. Since BIM is based on virtual models, it is challenging to fully reflect the dynamic and volatile nature of actual construction sites. Incorporating immediate changes occurring on-site into BIM models requires time and effort, which can result in delays for real-time monitoring. Moreover, although BIM provides information about construction sites through 3D visualization, it does not represent the actual site conditions as they are. Specifically, it has difficulties visualizing irregular and unpredictable on-site risk factors. Quantitative analysis shows that BIM-based monitoring systems experience an average delay of 15–20 s in real-time risk detection, which can make a critical difference in emergency situations. In conclusion, while BIM is a valuable tool for integrating and analyzing data related to construction site safety planning and knowledge management, it has limitations in reflecting real-time data and addressing the dynamic characteristics of site conditions. Therefore, it is necessary to integrate BIM with other technologies to complement these deficiencies.

In contrast, Point Cloud Data (PCD) registered through 3D laser scanning offers the advantage of visualizing three-dimensional spatial information that accurately reflects the actual site conditions. Unlike the virtual environment models of BIM, PCD includes data related to actual positions (x, y, z), intensity, and color (rgb) [13]. Quantitative research indicates that PCD-based monitoring demonstrates 98.3% accuracy in representing site conditions compared to BIM. However, existing PCD processing and visualization software has limitations in integrating various types of sensor data, and no approach has been proposed to utilize PCD for comprehensive construction site monitoring methods. The reason is that the data capacity of PCD has hardware limitations when operated in comprehensive visualization software [14].

Therefore, this study proposes a system that collects construction site data in real time using multiple sensors based on PCD and integrates and visualizes worker locations and concrete strength to implement a comprehensive smart construction monitoring system. The primary contributions of this study include the (1) development of a comprehensive monitoring framework that integrates multiple sensing technologies; (2) visualization of real-time data on an accurate PCD; and (3) empirical validation of the system’s effectiveness in enhancing construction site safety and quality management.

## 2. Literature Review

### 2.1. Sensor-Based Safety in Construction Site

Construction sites involve the simultaneous movement of various workers, machinery/equipment, and materials, with multiple tasks occurring concurrently. Due to this complexity, it is challenging to predict when and where risk factors may arise. Utilizing sensor technology to monitor dynamic elements in real time and detect hazards early can be highly beneficial. Safety managers and workers cannot always recognize and respond to all potential risks, and sensors can detect subtle changes that may go unnoticed by humans, thereby overcoming human limitations and ensuring a higher level of safety. Consequently, sensor technology is being employed to safely and efficiently monitor resources such as workers, equipment, materials, and structures on construction sites.

Real-Time Locating System (RTLS) refers to technology that tracks the location of objects or individuals in real time, utilizing various technologies such as GPS, RFID, Wi-Fi, Bluetooth, and UWB. In construction sites, RTLS technology can be employed to collect and analyze real-time location data of workers and equipment, enabling its application in various safety monitoring scenarios. According to several studies and statistics, the most common type of fatal accident on construction sites is falls from height [14,15]. RTLS technology can prevent such accidents by issuing warnings when workers approach fall-risk zones. Kiong et al. [16] reviewed the entire operational process of sensor modules and concluded that real-time location and situational awareness information could be used to prevent accidents and respond promptly to falls. Rey-Merchán et al. [17] evaluated the application of BLE monitoring systems for preventing falls from height during construction work and discussed methods for overcoming their limitations by integrating technologies such as GPS, BIM, and RFID.

RTLS technology is particularly useful for monitoring situations where workers and equipment are at risk of collision. Huang et al. [18] proposed a method using BLE-RTLS to track the locations of workers and equipment, effectively generating real-time warnings for workers in hazardous situations. Zhang et al. [19] conducted a study aimed at preventing workspace collisions by visualizing workspaces in BIM and categorizing them into worker spaces, material movement spaces, equipment/temporary structure spaces, and protective spaces (e.g., areas under cranes). They then collected GPS data for worker location tracking to detect collisions within workspaces. This study demonstrated that workspace occupancy parameters could be calculated using GPS data, enabling the detection of workspace congestion, fall risks, and collision hazards. However, it was noted that GPS data worked effectively only in outdoor environments, and alternative technologies such as UWB were required for indoor construction activities. Such advancements in RTLS have expanded its applications from outdoor positioning to indoor tracking on construction sites [20]. Nevertheless, there remains a lack of research discussing technologies and algorithms for indoor location tracking in construction environments [21]. Therefore, further research is needed to develop methods capable of seamless indoor and outdoor location tracking for safety monitoring in construction sites.

Temperature and humidity sensors are widely utilized on construction sites for protecting workers’ health, monitoring site conditions, and safeguarding materials and equipment. Construction workers are at a high risk of excessive heat exposure due to various factors. Shakerian et al. [22] demonstrated the potential of wristband-type biosensors to assess heat stress risks. Similarly, Jebelli et al. [23] evaluated workers’ physiological conditions by measuring skin temperature using wearable wristband sensors, enabling the early detection of stress factors. Additionally, Fang et al. [24] analyzed thermal parameters and workers’ heat responses to measure outdoor work environment temperature and humidity, recommending measures to reduce heat exposure risks to ensure workers’ safety during the summer. Temperature sensors are particularly useful for protecting materials and equipment. For example, concrete curing involves maintaining specific temperature and internal moisture conditions to achieve desired strength and durability. This process is highly sensitive to temperature and humidity, directly impacting concrete quality. In cases where poor quality results in reduced concrete strength, collapses may occur, causing significant human and economic losses. Jo et al. [25] utilized Fiber Bragg Grating (FBG) sensors to monitor the temperature and deformation of concrete during the curing process in real time. Similarly, Woldeamanuel et al. [26] proposed a method combining thermal imaging and deep learning-based image segmentation to estimate concrete strength in real time by extracting temperature data. These studies confirm that maintaining appropriate environmental conditions using temperature and humidity sensors can prevent material quality degradation and ultimately enhance structural safety.

Various sensors capable of detecting motion can also be utilized in construction. The Inertial Measurement Unit (IMU), which includes gyroscopes and accelerometers, is used to measure an object’s motion, such as position, velocity, acceleration, and rotation rates. Khan et al. [27] proposed a method using IMU sensors to acquire kinematic data and stable tilt angles of safety hooks to prevent fall-from-height (FFH) accidents. Their system alerts workers in real time when safety hooks are not attached to scaffolding, demonstrating the potential for IMU sensors to significantly reduce FFH accidents on construction sites with high accuracy and precision. IMU sensors can also be applied to monitor unsafe operations of construction machinery. Additionally, their capability to detect subtle movements makes them valuable for structural safety monitoring. Ragnoli et al. [28] used accelerometer-based sensor nodes attached to scaffolding and vertical structures to monitor tilt changes over time. Su et al. [29] proposed an IoT-based monitoring system to prevent the collapse of scaffolding shoring systems during concrete formwork construction. This system utilized wireless displacement, pressure, and angle sensors to monitor the instability of scaffolding during concrete formwork operations. Structural safety monitoring plays a critical role in preventing major accidents, such as structural collapses. The use of motion-detecting sensors enhances the ability to monitor and ensure safety in dynamic and high-risk construction environments.

### 2.2. Point Cloud Data for Construction Monitoring

PCD represents objects in 3D space as a collection of points, each with X, Y, and Z coordinates. It is typically generated using Structure from Motion (SfM) techniques based on 2D images or through 3D laser scanners equipped with LiDAR sensors. In particular, 3D laser scanners are widely used for obtaining geometric information of large-scale structures such as buildings, bridges, ports, and roads due to their ability to rapidly and accurately capture extensive datasets [30]. PCD generated by 3D laser scanners consists of spatial coordinates, color, and intensity information, creating a highly accurate digital twin of the actual environment. As PCD focuses on precisely reflecting real-world conditions, it is highly useful for site inspections and quality control to verify the construction status of existing structures. Due to these characteristics, PCD can be utilized for various purposes in construction site safety, such as structural safety and quality inspections, as well as tracking asset location information. Qureshi et al. [31] used PCD to evaluate the quality (e.g., rebar spacing, diameter) and quantity (e.g., rebar count, length) of rebar in construction sites, demonstrating over 99% accuracy for rebar count and length, 97% accuracy for spacing, and over 90% accuracy for diameter. Wang [32] proposed a technology to automatically verify scaffold and platform safety regulations using PCD to prevent scaffold-related fall accidents. The experimental results showed that the proposed method successfully extracted scaffold components and accurately identified safety violations in accordance with specified regulations.

Fang et al. [33] proposed a method for tracking the 3D locations of mobile assets (e.g., workers, equipment, materials) on construction sites by generating PCD from 2D images acquired using UAVs (Unmanned Aerial Vehicles). Their approach demonstrated that PCD could reconstruct the overall structure of construction sites, aiding in the visual analysis and understanding of real-world site conditions. This method addresses the limitations of 2D image-based vision studies, which lack 3D information. By leveraging this approach, it is possible to track not only the locations of assets but also changes across the entire construction site, with the added benefit of continuously updating the 3D site model to reflect ongoing site progress. However, the study relied on the SfM technique to generate PCD from UAV-acquired 2D images, which requires significant computation time, limiting its capability for real-time asset tracking. Furthermore, compared to PCD obtained via 3D laser scanners, the PCD generated using SfM lacks accuracy and density, making precise location tracking challenging. To overcome these limitations, integrating the location sensor technologies mentioned in Section 2.1 with PCD could improve both tracking accuracy and precision. However, integrating diverse sensor data into PCD is not a common practice. The primary reason is that most PCD processing and visualization software optimized for 3D geometric information lacks the functionality to integrate additional sensor data effectively [34]. For this reason, many studies have proposed construction site monitoring methods using BIM technology, an information model designed for managing and analyzing construction data, rather than relying on PCD for integrating various sensor data.

However, real-time construction site monitoring methods using BIM technology face several limitations. Hossain et al. [35] identified hazardous zones on construction sites and mapped them onto floor plans, integrating GPS-based mobile apps with cloud BIM to monitor unsafe worker behaviors in real time. The study noted that integrating BIM with other tools and IoT devices could enhance functionality. However, the authors highlighted that accurate definitions of hazardous zones and the geometric precision of BIM are crucial for system performance but are challenging to maintain consistently during ongoing construction. Park et al. [36] proposed a safety monitoring system integrating BLE-based location detection technology, BIM-based hazard identification, and a cloud-based communication platform. The study defined hazardous zones in BIM models either automatically or manually. The proposed system collected worker location data in real time and monitored their exposure to risks within the BIM environment, achieving a 97.5% detection rate for potential hazard situations in actual construction site tests. However, the system’s performance depended on the accuracy of the BIM model and hazard zone definitions. Discrepancies between the BIM model and actual site conditions due to ongoing construction necessitated manual adjustments to the zones.

A major limitation of BIM-based real-time monitoring is the difficulty of maintaining geometric accuracy as discrepancies arise between the BIM model and actual site conditions during construction. In contrast, PCD provides precise 3D coordinates of the actual environment, which can address this issue. By conducting safety and quality inspections based on high-precision PCD and by integrating diverse sensor data, the limitations of BIM-based sensor integration and 2D image-based construction site monitoring can be mitigated.

### 2.3. Previous Review

The previous studies [11,37,38,39] aimed to implement real-time construction site monitoring using smart technologies. The primary focus of these studies was to enhance construction site safety and productivity, support decision-making processes, and identify and manage risk factors proactively. However, several limitations have been identified.

The major issues include hardware and technical constraints, which can result in compatibility challenges among various sensors and devices, as well as reliability issues in data transmission. Limitations in data integration and management pose challenges in effectively processing and analyzing large volumes of data. Additionally, the cost and complexity of system development and operation hinder the adoption of technology on construction sites. Problems with scalability and applicability make it difficult to develop universal solutions adaptable to diverse site environments, while user interfaces and human factors influence user convenience and learning processes during system use. Noteworthy challenges include the technical complexity of integrating various smart construction elements into a single platform, cognitive overload for both on-site workers and managers, and the need for education and training to effectively utilize these technologies. The lack of systems capable of integrating diverse data for real-time construction site monitoring is another critical limitation. Existing systems often process specific datasets individually or provide fragmented monitoring, lacking the ability to analyze and visualize data comprehensively and in real time.

While BIM-based approaches have been actively explored, attempts to integrate PCD into such systems remain rare. BIM has established itself as a powerful tool with its 3D design and data-centric approach, but the application of PCD is still in its infancy, with technical and operational constraints hindering its integration into real-time monitoring systems. These limitations underscore the need for the development of systems that are practically applicable to construction sites. Future research should address these limitations and focus on developing user-friendly, efficient, and integrated smart construction systems. Such systems should incorporate tools for effective data analysis and visualization, lightweight hardware, and real-time decision support capabilities to ensure their practicality and adaptability in dynamic construction environments.

## 3. Methodology

### 3.1. System Concept and Configurations

**Figure 1:**
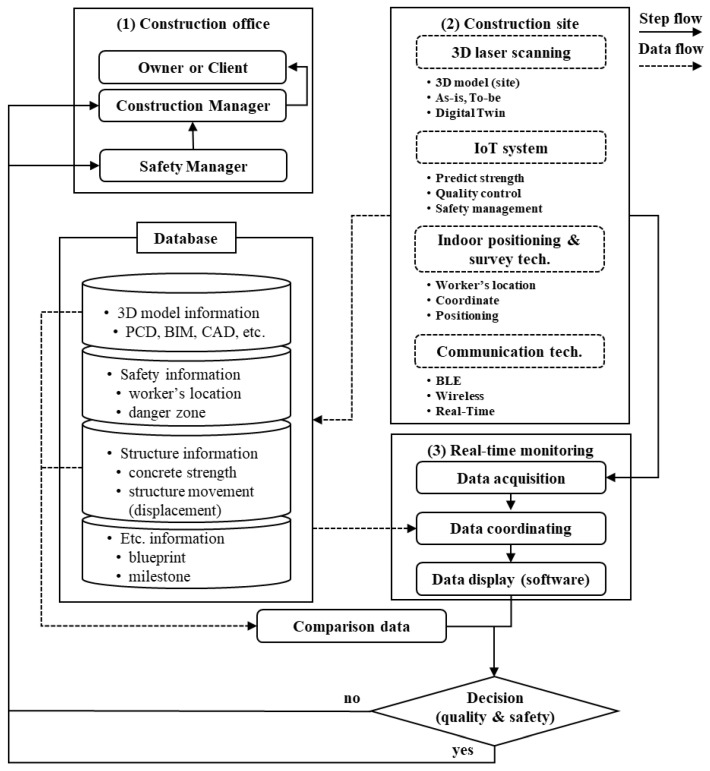
Overview of the real-time construction monitoring system.

This section provides an explanation of the various component technologies applied to the newly proposed real-time construction monitoring system for preventing industrial accidents on construction sites. Figure 1 illustrates an overview of the system. The system comprises several key component technologies, including 3D laser scanning for generating 3D models, indoor positioning, and surveying technologies for tracking workers’ locations and collecting site coordinate information, IoT for collecting concrete strength estimation data, and wireless communication technologies for transmitting and receiving data collected from construction sites. In the proposed process, element technologies are interconnected to monitor construction sites in real time and detect potential risk factors in advance, contributing to the quick and accurate management of sites by construction managers or safety managers by utilizing smart construction technologies. Detailed descriptions of each component technology and their applications will be discussed sequentially in the following subsections. This will provide a deeper understanding of the configuration and operational principles of the smart construction monitoring system.

### 3.2. System Component Technologies

#### 3.2.1. Three-Dimensional Laser Scanning Technology

**Figure 2:**
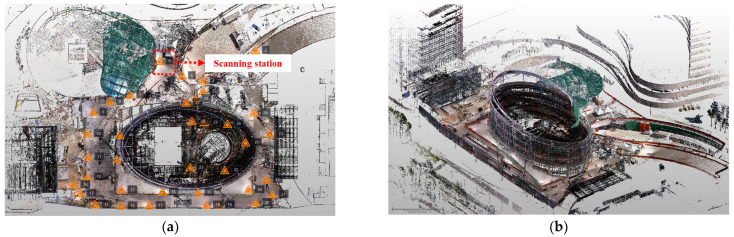
Example of PCD using 3D Laser scanning technology. (**a**) 3D laser scanning station. (**b**) Registration PCD.

Three-dimensional laser scanning technology has emerged as a critical component in construction automation, offering significant advantages in various aspects of project execution and management. This non-contact, non-destructive technology enables the rapid and precise collection of spatial data, providing a comprehensive digital representation of physical objects and environments [40]. In the context of construction automation, 3D laser scanning efficiently captures structural information from construction sites, including highly detailed geometric data of existing structures, ongoing construction, and surrounding environments. The resulting PCD is utilized for diverse purposes such as as-built documentation, progress monitoring, and clash detection [41]. Furthermore, the technology allows for the overlay of PCD with 3D design models, enabling accurate comparisons between design intent and actual construction to detect and resolve discrepancies at an early stage. High-resolution data captured by 3D laser scanners also serves as a valuable tool for quality control, ensuring compliance with design specifications and tolerances. Additionally, it provides a comprehensive record for future maintenance and renovation activities. In this study, 3D laser scanning technology was employed to construct a 3D model for visualizing information collected from various component technologies. As shown in Figure 2, the constructed PCD includes actual information from the construction site [13]. This model is used to visually identify workers’ locations and the manifestation of concrete strength in structures.

The integration of 3D laser scanning technology in this study provides a robust foundation for visualizing and analyzing critical construction data. By consolidating information from multiple sources into a comprehensive 3D model, project managers and stakeholders gain valuable insights into the progress and quality of construction activities [42]. This approach not only enhances the accuracy of project monitoring but also facilitates more informed decision-making throughout the construction process. Moreover, the application of 3D laser scanning in this context demonstrates its potential to improve safety measures on construction sites. By precisely mapping the locations of workers and equipment, it enables the early identification and resolution of potential hazards. Additionally, the ability to monitor concrete strength development in real time through visual representation offers significant advantages in quality control and ensuring structural integrity. In conclusion, the application of 3D laser scanning technology in this study underscores its versatility and importance in modern construction practices. By providing detailed, accurate, and easily interpretable representations of construction sites, this technology contributes significantly to improving project efficiency, safety, and overall quality within the construction industry.

#### 3.2.2. Worker Location Tracking

In this section, a fundamental methodological framework for indoor worker localization is briefly introduced to perform a qualitative assessment under static conditions. Detailed quantitative experimental results, including dynamic localization accuracy, real-time performance metrics, and comparative analyses among UWB, RTS, and 3D laser scanner, will be thoroughly presented in Section 4.2.

**Figure 3:**
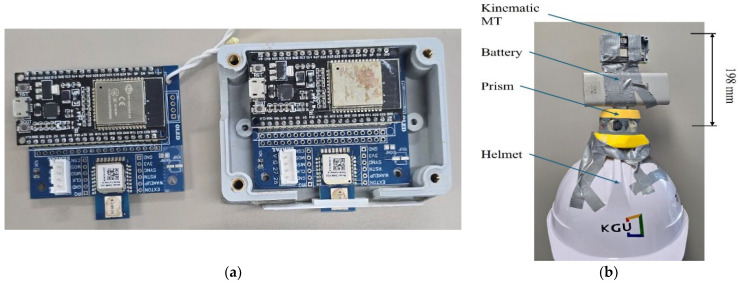
Overview of indoor worker localization system components. (**a**) DW1000 UWB sensor integrated with ESP32 Arduino board package. (**b**) Helmet equipped with UWB kinematic MT.

The proposed indoor localization system employs the DW1000 module based on Double Sided Two-Way Ranging (DS-TWR) UWB technology, which the manufacturer specifies as having a one-dimensional ranging accuracy of approximately 10 cm, consistent with previous studies [43]. Fixed UWB anchors were strategically installed at predefined reference points across the construction site, while a single UWB kinematic Mobile Terminal (MT) was attached to each worker’s helmet to track real-time positions (see Figure 3).

Given that anchor–MT geometry critically affects positioning accuracy, the layout was optimized following best-practice guidelines [44]. The UWB kinematic MT continuously acquired one-dimensional ranges to surrounding anchors via a Time Division Multiple Access (TDMA) schedule (50ms), at approximately 2–3 Hz. These range measurements were subsequently processed using a Python (ver. 3.12)-based 2D trilateration algorithm, performed at approximately 1 Hz.

For worker location tracking, an algorithm based on the code presented by Trojer [45], which utilizes three anchors, was improved. This modification was necessary as the construction site required the tracking of data for more than three workers. The algorithm was updated to allow the use of four or more positioning data points. In the original code, a master–slave configuration was implemented, where the MT communicated with one slave anchor to obtain ID and distance measurements before moving sequentially to the next slave anchor. This sequential methodology posed limitations in rapidly positioning kinematic targets. To address this, the system was reconfigured so that the MT attached to the worker’s helmet performed the role of the master anchor, while the remaining anchors acted as nodes. Consequently, the master–slave relationship was reversed, enabling faster communication during the UWB-based positioning process, and the number of ranging measurements was increased.

**Figure 4:**
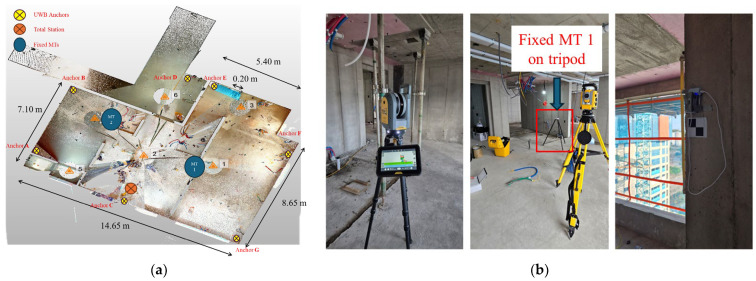
UWB sensor layout and sensor deployment at the construction site [43]. (**a**) Layout of UWB sensors illustrating anchor positions and MTs relative to structural elements. (**b**) On-site installation of TLS targets, RTS prism, UWB anchors, and black-and-white registration targets for accurate positioning.

Thus, the trilateration algorithm employed iterative linear least-squares computations, converging to a positional threshold of 5 cm within a maximum of 10 iterations, consistent with [43]. The relatively loose TDMA timing and sufficient measurement redundancy ensured stable real-time computations. Due to the presence of structural load-bearing walls, as illustrated conceptually in Figure 4a, maintaining full-floor line-of-sight (LOS) conditions would necessitate impractically dense anchor deployments. To mitigate this, elevation data derived from point-cloud models were utilized to fix the vertical coordinate (*z*-axis), effectively reducing the localization problem to planar (2D) trilateration and substantially lowering anchor density requirements.

All computations were performed locally to eliminate external network delays: an Arduino-based master node (UWB transceiver + 2.4 GHz Wi-Fi) transmitted raw ranging data to an on-site laptop, where real-time trilateration calculations were executed and integrated into the monitoring system. Baseline accuracy validation involved a Trimble RTS773 total station, automatically tracking a reflective prism attached to the worker’s helmet (Figure 3b) to supply precise ground-truth data. Additionally, two fixed reference UWB sensors were installed on tripods (Figure 4b) to perform static accuracy assessments. The results of these static accuracy evaluations under clear LOS and partial LOS conditions are detailed in Section 4.2.

#### 3.2.3. Predicting Concrete Compressive Strength

**Figure 5:**
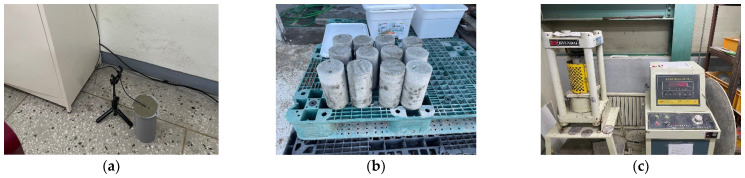
Overview of testing to build a concrete maturity model. (**a**) Measurement heat of hydration. (**b**) Concrete test specimen. (**c**) Compressive strength test.

To establish a reliable model for estimating concrete curing strength, we adopted the maturity-based strength prediction model from ASTM C 1074 [46] (Equations (1) and (2)) due to its proven accuracy and wide acceptance in the construction industry. A total of 15 specimens (3 per batch) were prepared using concrete applied on construction sites, and compressive strength measurements were conducted five times (at 1 day, 3 days, 7 days, 14 days, and 28 days). The results, along with the hydration heat data collected from two specimens, are presented in Table 1, as shown in Figure 5. For the hydration heat data, validated thermocouple sensors were used to collect concrete hydration heat at 1 h intervals. The concrete curing strength model, developed using the collected compressive strength and hydration heat data, is presented in Equations (3) and (4).

**Table 1:** Test results of concrete compressive strength and maturity.

Date (Month-Day)	Compressive Strength (MPa)				Maturity (°C-hrs)	Duration
	Specimen 1	Specimen 2	Specimen 3	Aver.		
11.23	4.39	7.15	7.18	6.24	733.58	Day 1
11.25	14.58	14.46	14.70	14.58	2246.64	Day 3
11.29	15.36	17.58	16.62	16.52	5147.7	Day 7
12.06	19.45	19.78	19.54	19.59	10,156.21	Day 14
12.20	25.17	27.33	26.49	26.33	20,296.52	Day 28

M = Σ(T – T0)Δt,(1)

where:

M = maturity index

T = average concrete temperature during the time interval Δt

T0 = datum temperature (usually taken as −10 °C)

Δt = time interval

S(t) = A + B log[M(t)],(2)

where:

S(t) = concrete strength at time t (Mpa)

M(t) = maturity at time t (°C-hrs)

A and B are curve coefficients specific to the concrete mix

y = 4.9977ln(x) – 26.4978 R^2^ = 0.9490, (3)

y = 11.5075log_10_(x) – 26.4978 R^2^ = 0.9490, (4)

#### 3.2.4. Real-Time Communication Technology

This study utilized the BLE method, known for its low power consumption and strong signal strength, to accommodate the communication environment of construction sites. The BLE method played a role in transmitting data collected at the construction site to the computer environment in real time. In this study, BLE was used to transmit internal and external temperature, humidity information, and construction worker location information to predict concrete strength at a construction site. For information on predicting concrete strength, the same information was collected for the measurement area where the sensor was embedded at the construction site. A BLE scanner collects this data and transmits it to a server via LAN, while a developed PC program accesses the server to compile the collected information. The reasons for employing BLE technology are as follows:

First, the energy-efficient characteristics of BLE extend the battery life of sensors, allowing long-term monitoring without frequent maintenance [47].

Second, the absence of wired connections simplifies installation and reduces the risk of communication infrastructure damage in constrained environments such as construction sites.

Third, BLE signals can penetrate various construction materials, enabling stable data transmission even in complex structural layouts [48].

Fourth, a system utilizing BLE can connect multiple beacons to a single scanner, making it applicable to large-scale construction sites.

Fifth, BLE is cost-effective compared to other communication methods, making it suitable for construction sites where numerous beacons are required.

Finally, BLE’s frequency-hopping spread spectrum (FHSS) helps maintain stable connections in construction sites with numerous electronic devices and potential interference sources.

By utilizing these characteristics of BLE, the goal is to collect information such as quality status checks on the progress of construction at construction sites, location information of workers, access to hazardous areas, etc., in real time, and display them visually on the PCD indicating the site status so that construction managers can manage them.

### 3.3. Data Mapping and Monitoring Based on PCD

In this study, methods such as PCD down-sampling and capacity reduction during the PCD output process were employed to display the 3D model (PCD) collected from a 3D laser scanner on a monitor. The reason why PCD is used instead of BIM in this study is that PCD reflects the actual condition with a high level of accuracy, and in this study, it is important to understand the current status according to the progress of construction, rather than understanding design information or future conditions like BIM. The detailed procedure is as follows.

First, the necessary monitoring model is created from the construction site. During this process, PCD from various locations on the site is collected using a 3D laser scanner, and 2D drawings or 3D models (Revit, SketchUp, etc.) are prepared based on design documents. Subsequently, PCD collected from various locations in the field are automatically registered to a common coordinate system through a registration process and converted to file formats (.las, .rwp, .e57) compatible with monitoring programs. In this study, Trimble’s Realworks 12.0 was used for alignment and file format conversion to perform automatic registration based on similar points. Each point in a PCD contains location, color, and intensity attributes, and the total number of points in a PCD can range from thousands to billions, significantly impacting file size. To down-sample PCD, either a point cloud thinning algorithm, which removes unnecessary points around the scanner, or a point cloud decimation algorithm, which reduces the number of points while maintaining the overall structure, is used.

The prepared PCD, along with the design-based 2D and 3D models, is then imported into the monitoring program. The imported data is saved as a default value in the program so that the existing model is displayed upon program restart. These imported models serve as the visual background for displaying concrete strength values and worker location information, as mentioned earlier. When diverse data are displayed in real time within the monitoring program, system overload may occur. To mitigate this, methods such as the Level of Detail (LOD) algorithm can be used. The LOD in PCD can be classified based on density, resolution, and abstraction level [49]. While LOD4 retains 100% of the original PCD density, enabling precise model analysis, its massive data size (e.g., 142,231,252 points, 5.3 GB, .las) poses challenges for real-time visualization. To address this, our study employs LOD3 (65,124,521 points, 1.7 GB, .las), which utilizes 50% down sampling to preserve structural outlines and ensure object identifiability while optimizing computational load. These techniques ensure that diverse information can be displayed in real time without overloading the system.

## 4. Filed Application

### 4.1. A Case Description

To validate the field applicability of the real-time construction monitoring system developed in this study, an empirical study was conducted at an actual construction site. This study took place at a residential building construction site from 14 October to 30 October 2024. The system was applied to specific floors to assess its normal operation under various variables present in construction sites and to evaluate the accuracy, stability, and visualization management capabilities of the smart construction monitoring system, including 3D laser scanning technology. Additionally, the study aimed to identify the system’s effectiveness and areas for improvement. Data were collected from two floors undergoing concrete pouring during the structural construction phase, and real-time monitoring was conducted. During this process, the predicted concrete strength at the time of formwork removal was checked for compliance with the standards specified in the architectural construction standard specifications. Furthermore, the system monitored whether workers approached hazardous locations. The building information for this empirical study is provided in Table 2.

The 3D laser scanner used for generating the PCD in this study was the Trimble X7 (Trimble Inc., Westminster, CO, USA), with detailed specifications provided in Table 3. To collect concrete strength information, thermocouple sensors (Analog Devices Inc., Wilmington, MA, USA, DS18B20) embedded within the concrete were employed. For measuring the concrete curing environment, temperature and humidity sensors (Sensirion AG, Stäfa, Switzerland, SHT31) were utilized, with detailed specifications outlined in Table 4. These sensors were selected due to their affordability and ease of acquisition, which was crucial for conducting experiments that required a large number of specimens for field application tests.

### 4.2. Practical Application and Results

**Figure 6:**
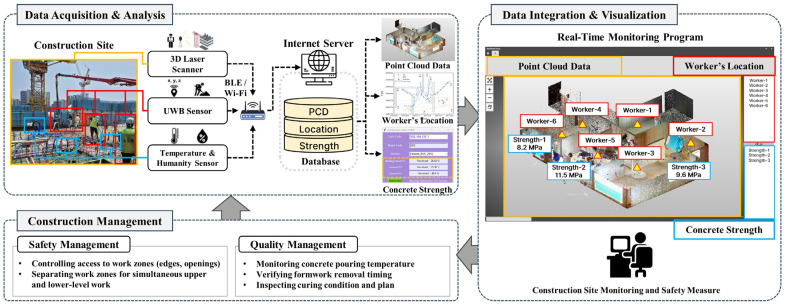
Integrated framework for real-time construction site monitoring system.

The smart construction monitoring system developed in this study is structured as shown in Figure 6. The system consists of three main stages: data acquisition/analysis, data integration/visualization, and safety and quality management. The image presented in the data acquisition section is of the upper floor where concrete pouring work is in progress, and sensors for predicting concrete strength are installed on the upper floor and the floor immediately below, while the image in the data integration section outputs the locations of workers checking the formwork status during concrete pouring on the lower floor.

The first stage involves collecting various data generated on construction sites and analyzing it in real time. At the empirical study site, data were acquired using a 3D laser scanner, UWB sensors, and temperature and humidity sensors. In order to create a 3D PCD model of the study site, a total of 42 station shots were taken using X7 (Trimble Inc., United States), and it took about 2 h to align and post-process the shot data using Trimble’s Realworks. In this study, data was collected for only one floor to verify the proposed system, and in an actual process, data for each floor can be collected and utilized as construction progresses. This enabled a clear visual identification of process status, structural geometry, and potential risks associated with ongoing construction work.

**Figure 7:**
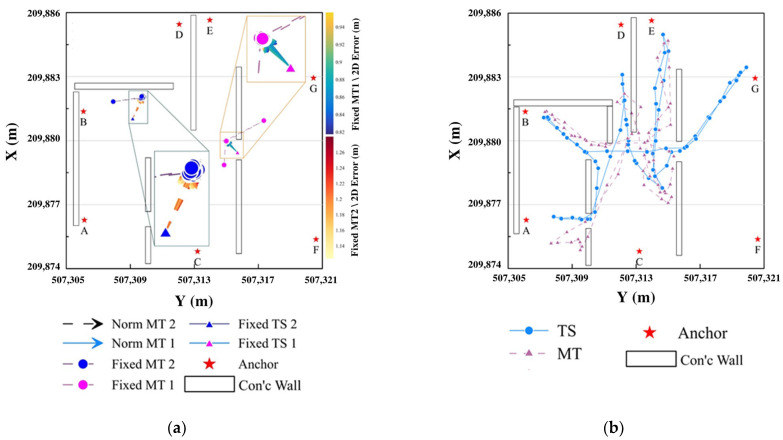
Analysis of the positioning results of kinematic MT at construction sites [43]. (**a**) Evaluating accuracy with fixed MTs. (**b**) Kinematic MT and RTS773 path analysis.

Static accuracy was evaluated using Fixed MTs placed on tripods at predefined locations (Figure 4). As shown in Figure 7a, under clear LOS conditions between anchors (B, C, E, F, G) and Fixed MT 1, the localization error ranged from approximately 0.80 to 0.94 m. Conversely, under partial NLOS conditions involving anchors A, B, and either C or D, the error increased by roughly 30 cm, yielding errors ranging from 1.14 to 1.26 m. These results emphasize the importance of maintaining LOS conditions and optimal geometric arrangement of anchors for enhancing localization accuracy. Moreover, Figure 7a clearly illustrates that different accuracy levels between MT 1 and MT 2 occur due to variations in LOS/NLOS conditions and geometric anchor–MT arrangements.

Dynamic accuracy assessments were performed by tracking the real-time positions of a worker equipped with a helmet-mounted UWB kinematic MT, continuously moving across the construction site. Real-time positions computed via UWB trilateration were compared against reference trajectories obtained from Trimble RTS773 total station measurements, as illustrated in Figure 7b. Results indicated a maximum localization error of approximately 1.44 m. Due to the RTS tracking system’s inherent delay (~2 s, 0.5 Hz), a perfect one-to-one comparison was challenging; however, these results confirm the UWB-based system’s sufficient accuracy for real-time worker tracking and safety management applications (Table 5).

**Figure 8:**
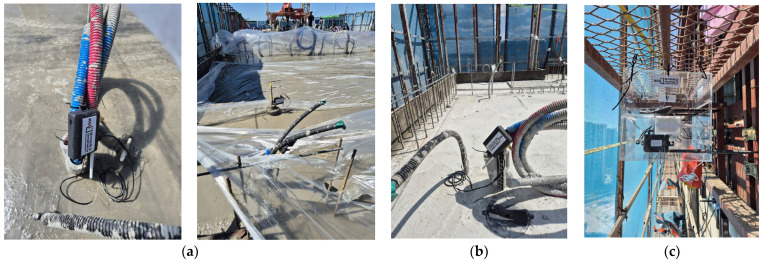
Application of the IoT system to predict concrete strength. (**a**) Sensor status at fresh concrete. (**b**) Sensor status after pouring. (**c**) Data communication system status at field.

As shown in Figure 8, the installed temperature and humidity sensors collected concrete curing strength data from two floors at hourly intervals. Using the concrete strength prediction model developed in this study (Equations (3) and (4)), the predicted concrete strength results were presented in Table 6 at hourly intervals. The data collected were transmitted to an external internet server via BLE or Wi-Fi. During this process, the sensor data were stored on a central server and processed in real time. The central server processed and analyzed the collected data in real time to generate critical information such as the status of the working environment, worker locations, and concrete strength.

**Figure 9:**
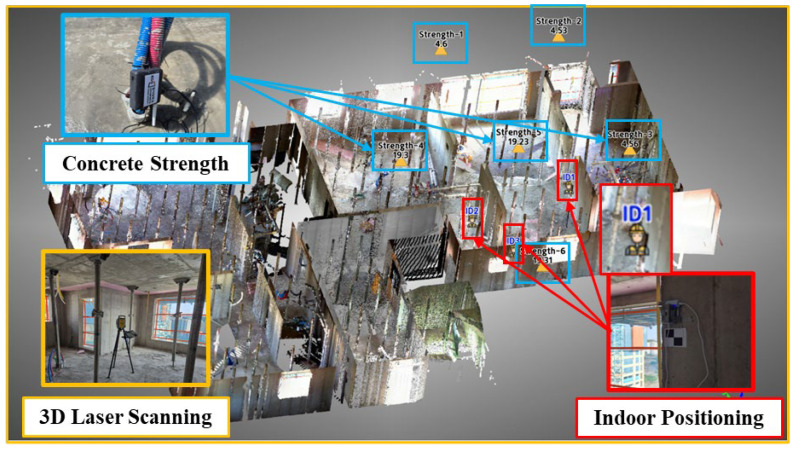
Real-time monitoring system preview.

The second stage involves integrative management of the collected data and visually representing it to support user understanding. PCD generated by the 3D laser scanner, worker location information, and concrete strength data are integrated and visualized through a real-time monitoring program, as shown in Figure 9. PCD of the construction site provides an intuitive representation of the actual working environment to the site manager, conveying the current site conditions effectively. Additionally, workers’ locations and movement paths are updated in real time, allowing the positions and related information for each worker to be easily identified. Concrete strength data are displayed by zone, enabling the status of strength development to be monitored and supporting decision-making processes such as determining the timing for formwork and shoring removal.

The third stage involves managing the safety and quality of the construction site based on the collected data. An example of safety management is controlling access within work zones to prevent workers from entering hazardous areas (e.g., edges, openings). However, in the demonstration presented in this study, visualization information related to the setting of risk zones was not output. The reason is that there is no risk zone in the area, and when a risk zone is actually set, an icon with an ID such as ID1 shown in Figure 9 is displayed in the risk zone of the PCD, and risk information is provided when the worker ID and the risk zone ID are close. An example of quality management is determining whether the design strength has been achieved and subsequently removing formwork and shoring at the appropriate time to ensure the quality of the structure.

### 4.3. Case Scenarios for Construction Site Monitoring

To evaluate the field application scenarios of the developed system, the safety and quality management status of the empirical study construction site was reviewed. The site was undergoing concrete pouring during the structural construction phase, coinciding with early winter conditions in Korea, characterized by gradually decreasing temperatures and significant daily temperature fluctuations. From a quality management perspective, key considerations included managing concrete hydration heat, ensuring the development of initial strength, and implementing effective curing plans. Additionally, measures were required to prevent cracking caused by rapid temperature changes and to protect equipment and materials from freezing. From a worker safety management perspective, aside from the mandatory use of basic safety equipment, additional management was necessary to prevent falls and collisions (e.g., access control to hazardous areas, use of safety nets) and to prevent slips on icy surfaces.

From a safety management perspective, the key monitoring items include control-ling worker access to edges and openings, separating upper and lower work zones, and managing the movement paths of equipment and vehicles. Real-time monitoring of workers approaching hazardous zones helps prevent “fall” accidents caused by workers failing to notice edges or openings in elevated areas. Additionally, access control to hazardous zones can prevent “struck by object” accidents caused by falling materials during simultaneous upper- and lower-level operations. For example, to prevent accidents from falling objects originating from upper-level work, work zones were segregated, and protective nets and covers were installed.

From a quality management perspective, the system enables the monitoring of the concrete-pouring temperature and material temperature, concrete hydration heat, the formwork removal timing, and the curing state and plan. At the empirical study site, real-time collection of material temperature data before concrete pouring ensured that the appropriate temperature was maintained. For instance, when the temperature in a specific zone dropped below the standard, heaters were activated immediately to maintain the temperature and prevent cracking. In winter, when concrete strength development slows, premature formwork removal can lead to structural collapse accidents. Since strength data are visually represented on the PCD, it is easy to verify at a glance whether the concrete strength at the time of formwork removal meets the criteria specified in the architectural construction standard specifications. If the required concrete strength has not been achieved, formwork removal can be postponed to prevent structural collapse risks.

Based on these theoretical contents and field test results, it is believed that further re-search implementation reflecting improvements will be necessary.

### 4.4. Results

The results of the empirical experiment confirmed that the developed system effectively collects various types of information from construction sites in real time and out-puts them to the monitoring program. The key findings are as follows:

Concrete strength prediction sensors and worker position sensors installed at construction sites transmitted data to the computer environment using BLE.

Worker location data were transmitted and visualized at 1 s intervals, enabling precise real-time path tracking with significantly improved responsiveness compared to conventional methods that typically operate at 5–10 s intervals. In the case of the risk zone, although it was not applicable in this study, the risk zone with the same ID as the worker and the worker ID were set to notify the manager of the dangerous situation through an alarm when they come near. This allowed for tracking workers’ movements and immediately identifying their access to hazardous zones.

For the information collected from the construction site for concrete strength prediction, the internal hydration heat generated during concrete curing and the external temperature and humidity for measuring the concrete curing environment were measured and transmitted in real time.

The predicted concrete strengths (average) were highly similar to the actual measured values, demonstrating the system’s accuracy:

Predicted strength at 14 days: 26.2 MPa (actual measured value: 25.3 MPa)
Predicted strength at 7 days: 19.3 MPa (actual measured value: 20.9 MPa)
Predicted strength at 1 day: 4.2 MPa (actual measured value: 4.8 MPa)

This enabled real-time monitoring of the concrete curing process and facilitated the timely progression to subsequent construction phases.

The PCD collected from the construction site using LiDAR collected information as the construction of each floor progressed, and in this study, a PCD for one floor was created to visually express the transmitted information in real time. At this time, in the case of the information collected for concrete strength prediction, information was collected every hour like the prediction model presented above and the concrete strength prediction was printed out. This enabled site managers to comprehensively understand the overall site situation and make prompt decisions. Additionally, the system detected and alerted potential safety risks, such as workers entering hazardous zones, in real time, which was evaluated as a significant contribution to accident prevention.

The review of the system’s construction site application scenarios confirmed its ability to prevent worker falls, object falling accidents, and collisions in terms of safety management. From a quality management perspective, it was found to help maintain material quality and prevent disasters such as structural collapses.

Compared to traditional manual construction site monitoring methods, the system developed in this study reduced the labor required by managers while providing insights into real-time risk situations from a macroscopic perspective.

These findings indicate that the developed system can be effectively utilized to improve the safety, quality, and productivity of construction sites. By collecting and analyzing data in real time, site managers were able to make quick and accurate decisions, which is expected to ultimately contribute to the successful execution of construction projects.

## 5. Discussion

A core aspect of this study involves utilizing 3D laser scanning technology to create and employ a PCD that most closely resembles the actual construction site for monitoring purposes. Traditional construction monitoring systems, which rely on 2D drawings or BIM, face limitations as they fail to reflect various site variables for real-time management. In contrast, the PCD used in this study captures the actual state of the construction site in real time with scanning frequency optimized at regular intervals to ensure continuous data collection for effective real-time management. This allows for more accurate and realistic monitoring of construction progress and safety hazards on site through a comprehensive time-synchronized data fusion mechanism that integrates all captured information within the same time interval.

This 3D model integrates various smart construction technologies, such as IoT sensors and worker location information collected from the construction site, to enhance the system’s functionality through a coordinated synergy of all captured data into a unified Smart Construction Monitoring System. By associating sensor data with specific locations in 3D space through standardized interfaces and protocols, the system can provide actionable, context-specific information that visually represents the real situation on the construction site. For example, the real-time monitoring system in this study incorporates concrete strength prediction technology, which can predict strength within 1–2 MPa of actual measured values as validated through extensive field testing. While this technology can be used independently for concrete quality management, the visual capabilities of the system enable it to predict curing strength at precise locations, contributing to more informed decision-making through a unified dashboard that presents all relevant data simultaneously. Similarly, the real-time indoor worker location tracking technology not only tracks the location of specific workers but also visualizes their positions within hazardous workspaces or zones on the 3D model through a robust edge computing architecture that ensures minimal latency in data processing and alert generation. This effectively identifies potential risks and helps reduce the occurrence of accidents by providing immediate notifications to both workers and supervisors when safety protocols are breached. By leveraging these smart construction technologies within a single integrated platform, it is possible to improve the working environment on construction sites and promote an overall enhancement of site safety culture, such as reducing accidents through proactive intervention rather than reactive measures.

While the integration of multiple technologies in the proposed system may present implementation challenges for small- and medium-sized construction companies, this can be addressed through modular system design, cloud-based services, and simplified user interfaces that reduce technical barriers and initial investment costs. These challenges stem from factors such as high initial investment costs, a lack of technical expertise, and maintenance issues that require appropriate technical support and training programs. However, small- and medium-sized construction companies often experience a higher incidence of safety accidents and fatalities due to weaker management structures and limited resources compared to large construction companies. This indicates that small-scale sites face lower efficiency in safety and quality management systems and have difficulties responding promptly to issues, making them ideal candidates for targeted implementation of smart monitoring solutions.

Therefore, smart construction monitoring systems should be prioritized for deployment at construction sites where safety management is limited, with implementation strategies tailored to the specific needs and constraints of smaller operations. Future smart construction monitoring systems should focus on simplifying implementation processes and reducing technical barriers to entry through plug-and-play components and standardized data exchange protocols that facilitate seamless integration with existing workflows. Additionally, user-friendly interfaces and intuitive data visualization tools should be developed to enable construction managers, who may not have prior exposure to smart construction technologies, to effectively utilize large volumes of data in their work through automated analysis and recommendation systems. Nevertheless, the expansion of these technologies is also necessary for large-scale construction sites where the complexity and scale of operations present unique challenges for comprehensive monitoring. For this, the system must be applied to larger sites beyond the scope presented in this study to collect extensive application data through pilot projects that demonstrate clear return on investment and operational benefits.

## 6. Conclusions

This study proposes a novel approach to real-time construction monitoring by integrating 3D laser scanning technology, IoT sensors, indoor positioning technology, and real-time communication technology into a single platform. The developed system is expected to serve as a supportive tool for construction site managers in tasks such as safety and quality management, thereby contributing to overall project efficiency. The application results of the smart construction monitoring system on construction sites are as follows:

1. The method proposed in this study accurately reflects the actual conditions of the construction site and can make a significant contribution to quality management.
2. By utilizing the location information of indoor workers at every second, it was possible to visually identify whether workers were approaching hazardous areas and potential risks during construction. This enabled managers to effectively contribute to safety management, even when they are not physically present at the site.
3. By providing hourly predictions of concrete strength, it was possible to estimate the strength of the concrete up to the point of formwork or support removal, thereby supporting both safety and quality management.
4. The real-time collection and analysis of data facilitated informed decision-making that was essential for construction operations.

The application of the smart construction monitoring system in this study verified its functionality and demonstrated the potential of smart construction technologies to revolutionize traditional construction management methods. By providing real-time, data-driven decision-making information, the system enables construction managers to make timely and informed decisions, reducing accidents, improving quality, and enhancing overall project outcomes.

However, this study has several limitations that could be addressed to develop a system with improved performance:

1. A key challenge identified in this study was the need to supply continuous power to sensor devices to enable real-time data collection and management. While this research addressed the issue by utilizing temporary power sources or auxiliary batteries, future studies should consider integrating small built-in batteries. This approach would help reduce the overall weight of the devices and enhance their usability.
2. The collection of on-site worker location data raises potential privacy concerns. In this study, since the verification process focused on concrete pouring tasks within structural works, it was not necessary to identify individual workers. However, a limitation remains: to fully implement the proposed technology on construction sites, it will be necessary to develop methods for identifying and classifying workers across various job types.
3. Successful on-site application of the proposed technology requires both site organization and the cooperation of site managers. Currently, site managers tend to have a negative perception of smart construction technologies, primarily due to the additional costs and manpower required for their implementation, especially given the limited adoption of such technologies, to date.
4. To implement the proposed system at construction sites, it is necessary to examine the feasibility of adoption by small and medium-sized construction companies through a cost–benefit analysis associated with system implementation and operation.
5. This system was applied to construction sites for a specific period, and its efficacy was verified by analyzing the data collected. However, for practical application in actual construction sites, further research is required on the analysis of application effects, such as the rate of safety incident reduction and the degree of quality improvement, as well as studies on productivity enhancement methods through long-term monitoring of the system.

In conclusion, the smart construction monitoring system presents a significant ex-ample of digitalization and automation on construction sites. Its successful implementation demonstrates its potential to contribute to high-quality construction projects. As the construction industry continues to evolve, innovative technologies are expected to meet the increasing demands for safety, efficiency, and sustainability in construction practices.

## Figures and Tables

**Table 2:** Building information for empirical study.

Location	Gyeonggi-Do, Republic of Korea
Number of Floors	2 basement and 30 floors (956 households)
Building Usage	Residential apartment complex
Site Area	63,239.60 m^2^
Total Floor Area	147,998.24 m^2^

**Table 3:** Specification of 3D laser scanner.

EDN laser class		Laser class 1, IEC EN60825-1
Speed		Up to 500 kHz
Distance		0.6~80 m
Time		2~15 min
Range	Accuracy	2 mm
	Noise	<3 mm @ 60 m on 80% albedo
	3D Point	2.4 mm @ 10 m/3.5 mm @ 20 m

**Table 4:** Specification of sensors using IoT system.

Specification	DS18B20	SHT31
Sensor Type	Digital Temperature Sensor	Digital Temperature and Humidity Sensor
Temperature Range	−55 °C to +125 °C	−40 °C to +125 °C
Humidity Range	None	0% to 100% RH
Temperature Accuracy	±0.5 °C (−10 °C to +85 °C)	±0.2 °C
Humidity Accuracy	None	±2% RH
Resolution	9 to 12 bits (user-configurable)	Temperature: 0.015 °C, Humidity: 0.01% RH
Supply Voltage	3.0 V to 5.5 V	2.4 V to 5.5 V
Interface	1-Wire	I2C
Response Time	750 ms (at 12-bit resolution)	Temperature: 2 s, Humidity: 8 s
Etc.	Available (TO-92 package)	-

**Table 5:** The distance from the UWB Anchors received by the kinematic MT and the resulting calculated coordinates (European Petroleum Survey Group (EPSG):5186).

Timestamp	ID	UWB Anchors to Kinematic MT Distance (m)							Calculated MT Coordinate (m)		Iteration
		Range A	Range B	Range C	Range D	Range E	Range F	Range G	X	Y	
11/06/2024 15:43:51	ID2	0	0	5.24	0	0	6.56	6.83	507,315.32	209,879.49	2
11/06/2024 15:43:52	ID2	0	9.98	4.11	0	8.51	0	0	507,315.38	209,878.49	3
11/06/2024 15:43:53	ID2	0	9.50	3.25	0	0	0	8.44	507,315.13	209,877.52	3
11/06/2024 15:43:54	ID2	0	9.57	0	0	8.7	0	9.17	507,314.36	209,877.01	2
11/06/2024 15:43:57	ID2	0	0	2.98	0	9.26	0	9.61	507,313.25	209,876.91	6
11/06/2024 15:43:57	ID2	0	9.00	3.05	0	0	0	9.55	507,312.95	209,877.20	3
11/06/2024 15:43:58	ID2	0	0	3.05	8.82	0	0	9.5	507,313.17	209,877.24	4
…											
11/06/2024 15:45:32	ID2	0	0	5.84	0	0	6.55	5.86	507,315.58	209,879.76	2
11/06/2024 15:45:34	ID2	0	0	5.98	0	0	6.36	5.72	507,315.80	209,879.86	2
11/06/2024 15:45:37	ID2	0	0	0	0	6.83	6.21	4.4	507,316.49	209,879.93	3
11/06/2024 15:45:38	ID2	0	0	0	0	6.6	6.54	3.71	507,317.32	209,880.33	3
11/06/2024 15:45:39	ID2	0	0	0	0	6.5	6.87	3.14	507,317.79	209,880.92	3
…											
11/06/2024 15:48:02	ID2	0	10.13	3.04	0	8.64	0	0	507,315.21	209,877.36	2
11/06/2024 15:48:03	ID2	0	9.80	0	0	8.45	0	8.29	507,315.00	209,877.08	2
11/06/2024 15:48:04	ID2	0	0	3.63	0	8.54	0	8.59	507,314.60	209,877.26	6
11/06/2024 15:48:05	ID2	0	0	4.35	0	8.25	0	8.8	507,313.49	209,877.92	6
11/06/2024 15:48:05	ID2	0	8.51	4.59	0	7.37	0	0	507,313.44	209,878.58	4
11/06/2024 15:48:07	ID2	0	7.41	4.82	6.78	0	0	0	507,313.81	209,879.00	3
11/06/2024 15:48:08	ID2	7.94	0	5.60	5.86	0	0	0	507,313.26	209,879.64	2
11/06/2024 15:48:10	ID2	8.18	0	6.09	5.1	0	0	0	507,313.16	209,880.36	2

**Table 6:** Result of measurement IoT system (concrete strength).

Timestamp	Device	Acquired Data from Construction Site			Calculation		Remark
		Temperature (°C)		Humidity (%)	Maturity (°C-hrs)	Strength (MPa)	
		Inside	Outside	Outside			
14/10/2024 08:27:45	255, 6	27.76	23.54	60.34	39.76	−8.09	Day 0
14/10/2024 09:28:47	255, 6	31.21	24.51	57.11	78.56	−4.69	Day 0
15/10/2024 08:28:50	255, 6	26.24	25.3	54.52	868.68	7.32	Day 1
15/10/2024 09:28:52	255, 6	26.02	24.8	54.5	904.58	7.52	Day 1
16/10/2024 08:31:50	255, 6	25.46	25.47	57.25	2582.26	12.77	Day 3
16/10/2024 09:32:52	255, 6	25.38	25.3	56.61	2617.39	12.83	Day 3
18/10/2024 08:36:53	255, 6	26.78	25.97	50.88	4260.53	15.27	Day 5
18/10/2024 09:37:06	255, 6	26.87	26.01	49.95	4295.61	15.31	Day 5
20/10/2024 08:39:11	255, 6	23.38	22.86	49.28	5967.35	16.95	Day 7
20/10/2024 09:39:14	255, 6	23.46	23.03	48.7	6003.42	16.98	Day 7
25/10/2024 08:40:12	255, 6	24.74	22.28	50.38	9430.36	19.24	Day 12
25/10/2024 09:41:05	255, 6	24.82	22.07	49.95	9466.11	19.26	Day 12
30/10/2024 08:45:47	255, 6	23.8	24.98	55.9	13,718.89	21.11	Day 17
30/10/2024 09:46:06	255, 6	23.2	25.43	55.38	13,754.07	21.13	Day 17

## Data Availability

The data presented in this study are available on request from the corresponding author due to legal reasons.
